# Surgical complications of radical retropubic prostatectomy: A single institutional experience of seven years

**DOI:** 10.4103/0970-1591.36708

**Published:** 2007

**Authors:** Neeraj K. Goyal, Abhay Kumar, Sameer Trivedi, Udai Shanker Dwivedi, Pratap Bahadur Singh

**Affiliations:** Department of Urology, Institute of Medical Sciences, Banaras Hindu University, Varanasi - 221 005, India

**Keywords:** Carcinoma prostate, radical prostatectomy

## Abstract

**Aim:**

To determine the surgical complications of open retropubic radical prostatectomy.

**Materials and Methods:**

Fifty-nine cases of localized prostate cancer underwent retropubic radical prostatectomy in our department in the last seven years. Standard technique of open retropubic radical prostatectomy as described by Walsh was used. During follow-up cancer control and quality of life indices (potency and urinary continence) were noted.

**Result:**

Postoperative recovery of all patients except one was excellent. This patient required cardio-respiratory support and nine units of blood transfusion. Forty-nine out of 52 patients were continent, two had stress incontinence and one was totally incontinent at one year. Bladder neck contracture was present in eight out of 52 patients at one year. Forty-five patients were impotent at one year with or without oral tablet sildenafil. Cancer control was present in 45 out of 52 cases. Seven cases had biochemical failure at one year.

**Conclusion:**

Though retropubic radical prostatectomy is the standard treatment for early prostate cancer it is not without complications. It has a steep learning curve. More number of cases and refinement in technique is required to achieve world-class results.

For organ-confined disease in carcinoma of prostate, the various treatment options are watchful waiting, radiation therapy or retropubic radical prostatectomy. Out of these, retropubic radical prostatectomy has been established as the primary curative procedure.[1,2] It is the most frequently performed procedure and is performed in 52% of patients, followed by external radiotherapy or brachytherapy used in approximately 20% of patients.[1] Even though it is the most commonly performed procedure, it is not without complications. It has been stated that the rate of complication is less with the other treatment modalities. We present this paper to highlight the various complications of the surgical procedure.

## MATERIALS AND METHODS

Our institute is a tertiary healthcare referral center. From February 2000 to February 2007, 291 cases of carcinoma of prostate were managed at our institution. Out of these, 129 cases were locally advanced prostate cancer and 82 had distant metastasis at the time of presentation. They were managed with hormonal derivation therapy. Eighty cases were of localized prostate cancer, out of which 59 were candidates for open retropubic radical prostatectomy. Written consent was taken in all these cases. All cases were operated by a single surgeon. Out of 59, seven cases were lost to follow-up and were excluded from the study. All patients were evaluated thoroughly by history and physical examination. Patients with lower urinary tract symptoms, above the age of 50 years and life expectancy more than 10 years were advised serum prostate specific antigen (S.PSA). If the S.PSA was more than 4ng/ml or digital rectal examination was abnormal, patients were advised for prostate biopsy. Then hematologic and radiologic investigations were done to rule out metastatic disease. Chest X-ray and ultrasound of whole abdomen was done. We routinely did MRI of pelvis to exclude loco-regional extent and presence of pelvic lymphadenopathy. Bone scan was done only if S.PSA was >10 ng/ml or Gleason grade was >7. All patients were operated with standard technique of radical retropubic prostatectomy described by Walsh.[2] Incision was midline, extraperitoneal extending from the pubis to the umbilicus. Bilateral pelvic lymph node dissection was performed. Extent of dissection was superiorly up to bifurcation of iliac vessels, inferiorly up to femoral canal, laterally up to external iliac vein and medially up to obturator vessels. Then bilateral incision in the endopelvic fascia, division of puboprostatic ligaments, division of dorsal vein complex, division of urethra, posterior dissection and division of lateral pedicles, division of bladder neck and excision of the seminal vesicle was done. The specimen was removed. The last step in the surgery was bladder neck reconstruction and anastomosis with urethra with 4/0 polyglactin over a silicon Foley catheter (16 French). A suction drain was kept in the retropubic space.

In the postoperative period, patients were ambulated in the next morning after the procedure and allowed liquids orally. Semisolid diet was given on postoperative day (POD)-2 and normal diet on POD-3. Drain was removed when it was less than 20ml per day (usually POD-3). Patients were discharged with Foley catheter in situ on POD-8 after removal of skin stitches. All patients were advised to get pericatheter study on POD-21. If there was no extravasation at the anastomotic line then voiding trial was given. If there was extravasation then Foley catheter was kept for six weeks. All patients were followed up three-monthly for the first two years, biannually for the next one year, annually thereafter. At every visit, patients were asked for symptoms if any, examined especially with per rectal examination, advised uroflometry and S.PSA.

## RESULTS

Patients profile has been shown in Table 1. Patients were proved to have adenocarcinoma of prostate with preoperative needle biopsy of the prostate. All patients except one had excellent postoperative recovery. This patient had adult respiratory distress syndrome and persistent bleeding from the drain till POD-9. He could be resuscitated with cardio-respiratory support in the intensive care unit and total of nine units of blood transfusion. Postoperative results are shown in Table 2. Forty-five patients were fully continent and seven patients were incontinent at one month. Pelvic floor exercises were taught to the incontinent patients. At one year, 49 patients were continent, two had stress incontinence and one was totally incontinent. Forty-five patients lost potency after the surgery. Eight patients had mild anastomotic site stricture in the follow-up period. Out of these, six patients responded well with one dilatation. Two patients required bladder neck incision.

**Table 1:** Patients profile

Mean Age in years (range)	61 (50–73)
IPSS Score	Number of Patients
Mild	7
Moderate	35
Severe	10
Abnormal DRE (n)	12
Preoperative S.PSA (ng/ml)	Number of Patients
<4	0
4–10	39
10–20	12
>20	1
Periprostatic extention in MRI (n)	1
Gleason's Score	Number of Patients
4	17
6	19
8	15
9	1

**Table 2:** Postoperative incontinence

Numbers of patients incontinent after catheter removal			
	At 1 month	At 6 months	At 12 months
Total incontinence	2	2	1
Mixed (stress and urge) incontinence	3	0	0
Stress incontinence	2	4	2
Continence	45	46	49
Total	52	52	52

## DISCUSSION

Carcinoma of prostate is the fourth most common male malignancy worldwide with the highest incidence in North Americans and Scandinavians, especially in African Americans (272 cases per 100,000). The incidence is the lowest in the Asian population (1.9 cases per 100,000).[3] There was no single standard treatment for localized carcinoma of prostate. Patient had various options like watchful waiting, radiation therapy or surgery. Nowadays radical retropubic prostatectomy (RRP)has been established as the standard treatment for localized carcinoma of prostate. Open surgical approach has been challenged by the advent of laparoscopy and techniques but the open procedure is still a “gold standard” for cancer control and quality of life.[4] Technically, it is one of the most difficult procedures in urology and not without significant complications. The three goals to be achieved during this surgery are cancer control, maintenance of urinary continence and lastly preservation of sexual function. Significant complications are intraoperative or postoperative bleeding, thrombo-embolism and loss of any goals as described above. But with better understanding of the pelvic anatomy, good surgical skills and ongoing experience in the selection of candidates, these complications have come down to an acceptable level.

In India as the incidence of carcinoma of prostate is low, the number of cases is less as compared to the west. So the Indian data on this disease is sparse. Only a few centers are performing radical retropubic prostatectomy because of limited number of cases and limited experience with the surgical technique. We present our experience with surgical technique, its complications and results. Though our experience is limited this article will have a message for Indian urologists to make it the first line therapy in localized prostate cancer. Initially, we were able to control cancer and continence but not able to preserve the potency in most patients. We haven't performed bladder neck sparing or seminal vesicle sparing RRP for the preservation of potency as we are not finding the appropriate candidates for it. We haven't come across any study in the Indian literature that has performed such procedures.

In the postoperative complications, incidence of significant postoperative hemorrhage is 0.5%[5] but in our series, it is one out of 52 (1.9%). We managed the patient conservatively. We did not find increased incidence of bladder neck contracture and incontinence in this patient as the patient was continent at one month and no bladder neck contracture was present.

Incidence of bladder neck contracture is 0.5-10%. In our series, the incidence of bladder neck contracture was eight out of 52 cases (15.4%). Two patients underwent bladder neck dilatation thrice without success. We did bladder neck incision with Collin's knife and patients are doing well. Mild contracture was present in six patients, which required single dilatation.

Urinary incontinence is usually secondary to intrinsic sphincter deficiency. Stanford *et al*.,[6] in 2000 reported an incontinence rate of 8.4%, but with introduction of bladder neck intussusception 98% of the patients were pad-free at one year.[7] In our series, 49 out of 52 patients (94.3%) were continent, two (3.8%) had occasional stress incontinence and one (1.9%) had total incontinence at one year. Patients were advised pelvic floor exercises and tablet Imipramine to help improve continence.

Regarding preservation of erectile function, three important factors are preoperative potency, age of the patient (less than 65 years) and ability to preserve neurovascular bundles. Walsh *et al*.,[8] in 2000 evaluated the sexual functions by a validated questionnaire and found that 86% of the patients were able to achieve erections with or without sildenafil citrate at 18 months. Parsons *et al*.,[9] in 2004 reported 42% recovery of erectile dysfunction at three months, 49% at six months and 73% at one year. In both series bilateral neurovascular bundles were preserved. Potency rate was 65% in whom only one neurovascular bundle was preserved. Unfortunately 45 of our patients became impotent after RRP, irrespective of preoperative potency status. This is because we were not able to preserve both neurovascular bundles during surgery. Recently, we started performing nerve-sparing RRP and were able to save erectile function. In the last seven cases we were able to maintain potency with the help of oral Sildenafil.

In conclusion, open radical retropubic prostatectomy is curative in early prostate cancer. The only concern is to select the appropriate candidates and the need to improve quality of life after the procedure.

Our results demonstrate the safety and efficacy of RRP in carefully selected patients. The aggressive approach for organ-confined disease in early carcinoma of prostate offers these patients an excellent chance of cure with good quality of life.
